# Supplementary material for: A Likelihood Approach for Real-Time Calibration of Stochastic Compartmental Epidemic Models
Source: PLoS Comput Biol. 2017 Jan 17;13(1):e1005257. doi: 10.1371/journal.pcbi.1005257 (PMC5240920; doi:10.1371/journal.pcbi.1005257)
Supplement: S1 File — (TAR.GZ) [file pcbi.1005257.s014.tar.gz › HSPH_Online-SI-Revision/Additions/MSS11/n100-extreme-SEITR/n100-extreme-SEITR_NEW_summary.pdf]

peak -8	R0	$1/\theta_2$	num week	inf	inflog	newly	newlylog	Reff	AttackRate
mean	30.6	44.2	256.2	47.5	0.3	69.1	0.3	30.7	25.3
median	24.4	37.6	100.5	47.9	0.3	45.8	0.2	24.4	17.8
Quantile .1	12.7	21.9	70.	25.8	0.1	15.	0.1	12.7	8.7
Quantile .9	57.5	69.6	554.	65.4	0.5	87.8	0.8	57.6	44.5
37	27	3							
length	50								

peak -4	R0	$1/\theta_2$	num week	inf	inflog	newly	newlylog	Reff	AttackRate
mean	22.6	46.7	136.4	46.7	0.3	10.9	0.	23.2	22.8
median	20.9	48.2	100.	47.3	0.3	7.5	0.	21.6	21.4
Quantile .1	6.2	21.	40.	20.8	0.1	4.1	0.	7.9	6.5
Quantile .9	38.1	70.8	236.	71.9	0.6	22.2	0.1	36.9	40.9
19	6	0							
length	50								

peak	R0	$1/\theta_2$	num week	inf	inflog	newly	newlylog	Reff	AttackRate
mean	25.1	36.6	914.3	40.6	0.2	14.4	0.1	191.1	11.9
median	15.1	40.8	100.	43.8	0.3	15.2	0.1	173.6	11.4
Quantile .1	2.9	12.8	0.	20.5	0.1	6.8	0.	110.2	3.9
Quantile .9	65.2	51.5	3820.	56.	0.4	21.3	0.1	301.9	18.9
1	0	0							
length	50								

peak +4	R0	$1/\theta_2$	num week	inf	inflog	newly	newlylog	Reff	AttackRate
mean	46.4	28.3	611.2	60.6	0.6	50.7	0.5	381.1	4.2
median	31.8	27.7	100.	62.3	0.4	47.5	0.3	297.7	4.2
Quantile .1	9.7	4.2	0.	19.6	0.1	22.	0.1	86.3	1.5
Quantile .9	89.3	45.3	1900.	99.6	1.1	89.5	1.	750.2	7.4
0	0	1							
length	50								

peak +8	R0	$1/\theta_2$	num week	inf	inflog	newly	newlylog	Reff	AttackRate
mean	47.7	32.9	431.	106.7	1.7	97.2	1.7	194.6	1.3
median	33.1	29.7	100.	99.5	1.8	99.4	1.9	112.2	1.
Quantile .1	9.9	4.8	0.	24.4	0.1	40.3	0.2	72.5	0.1
Quantile .9	90.1	63.4	900.	99.9	2.8	99.9	2.8	452.1	3.2
0	0	0							
length	50								
